# Serum Defensins in Differentiating Idiopathic Granulomatous Mastitis from Breast Cancer—Defensins in Idiopathic Granulomatous Mastitis and Breast Cancer

**DOI:** 10.3390/jcm15103660

**Published:** 2026-05-10

**Authors:** Berrin Papila, Naile Fevziye Misirlioglu, Emine Yildirim, Hafize Uzun

**Affiliations:** 1 Department of General Surgery, Cerrahpasa Faculty of Medicine, Istanbul University-Cerrahpasa, 34320 Istanbul, Türkiye; 2 Department of Biochemistry, Faculty of Medicine, Istanbul Atlas University, 34403 Istanbul, Türkiye; 3 Department of General Surgery, Faculty of Medicine, Istanbul Atlas University, 34403 Istanbul, Türkiye

**Keywords:** idiopathic granulomatous mastitis, breast cancer, defensins, α-defensin 1, β-defensin 1, β-defensin 2, differential diagnosis

## Abstract

**Background/Objectives:** Differentiating idiopathic granulomatous mastitis (IGM) from breast cancer (BC) remains a significant clinical challenge due to overlapping clinical and radiological features. This study aimed to evaluate the diagnostic value of serum defensins and conventional tumor markers in distinguishing BC from IGM. **Methods:** A total of 150 participants were included: 50 with BC, 50 with IGM, and 50 with healthy controls. Serum levels of α-defensin 1, β-defensin 1, and β-defensin 2 were measured and compared across groups. In addition, inflammatory markers and tumor markers were analyzed. Receiver operating characteristic (ROC) analysis and logistic regression models were used to assess diagnostic performance. **Results:** Serum defensin levels were significantly higher in BC and IGM compared to healthy controls (*p* < 0.001), with β-defensin 2 showing the highest levels in BC. ROC analysis demonstrated high diagnostic accuracy for defensins (AUC: 0.95–0.99); however, in multivariable analysis, defensins were not retained as independent predictors, whereas CA15-3 and CA125 remained significant. The combined model based on CA15-3 and CA125 showed good discriminative performance (AUC = 0.83). **Conclusions:** Defensin levels, particularly β-defensin-2, were highest in BC and showed promising diagnostic potential; however, they should be considered adjunctive biomarkers. Their integration with conventional tumor markers and inflammatory parameters may improve differentiation between BC and IGM.

## 1. Introduction

Idiopathic granulomatous mastitis (IGM) is a rare, benign chronic inflammatory breast disease characterized by non-caseating granulomas and abscess formation. Its clinical presentation closely mimics breast carcinoma. Since its first description in the 1970s, IGM has increasingly been recognized as a challenging diagnostic entity. This is largely because its imaging findings and clinical manifestations often overlap with those of breast cancer (BC) [1]. Differentiating IGM from malignancy is of utmost clinical importance, as management strategies differ significantly. IGM is primarily treated with corticosteroids or immunomodulatory therapy, whereas BC requires surgical and oncologic interventions [2].

BC remains the most common malignancy among women worldwide and is now known to be influenced by inflammatory processes. Elevated inflammatory mediators, including C-reactive protein (CRP), interleukin-6, and tumor necrosis factor-α, contribute to tumor initiation, progression, and metastasis. High CRP levels have also been associated with poor prognosis [3,4]. Conventional tumor markers such as carcinoembryonic antigen (CEA), CA15-3, CA19-9, and CA125 are widely used in clinical practice. However, their diagnostic performance in differentiating malignant tumors from benign inflammatory conditions remains limited. This overlap highlights the need for novel biomarkers with improved sensitivity and specificity [5].

Defensins are antimicrobial and cytotoxic peptides that play a key role in host defense. Beyond their role in innate immunity, they have also been implicated in various cancers, including colorectal cancer (CRC), BC, and kidney cancer [6,7]. Structurally, defensins are classified into α-, β-, and θ-subtypes [6,7,8]. In mammals, α-defensins function as potent mucosal antimicrobial peptides. Notably, α-defensin-1 has been reported to be overexpressed in BC patients achieving remission after paclitaxel therapy [9]. Human β-defensin-1, produced by neutrophils and epithelial cells, provides protection against microbes and toxins [10,11]. In contrast, β-defensin-2 exhibits broad antimicrobial activity and enhances immune responses [12,13,14,15].

The present study provides several novel contributions to existing literature. First, to the best of our knowledge, this is the first study to comprehensively compare circulating α-defensin 1, β-defensin 1, and β-defensin 2 levels across patients with BC, IGM, and healthy controls within a unified diagnostic framework. Second, beyond demonstrating group differences, we evaluated the diagnostic performance of these biomarkers using receiver operating characteristic (ROC) analysis and integrated modeling approaches. This allowed us to move beyond descriptive findings toward clinically relevant risk stratification. Third, β-defensin 2 demonstrated the highest discriminatory capacity with minimal overlap between BC and IGM. These findings suggest a potential adjunctive role for defensins, particularly in combination with established tumor markers, in challenging differential diagnoses.

## 2. Materials and Methods

### 2.1. Ethical Approval

This study received approval from the Ethics Committee of Istanbul Atlas University Medical Faculty (protocol number: E-22686390-050.99-73404. Date: 5 August 2025), and written informed consent was obtained from all participants. The study was conducted in compliance with the Declaration of Helsinki.

### 2.2. Study Design and Participants

A total of 150 participants were included: 50 with BC, 50 with IGM, and 50 with healthy controls. BC patients were newly diagnosed and had not yet undergone surgical intervention, chemotherapy, or radiotherapy. Patients with IGM were diagnosed based on core needle biopsy findings showing non-caseating granulomatous inflammation in the absence of infectious or systemic granulomatous diseases. Exclusion criteria for both patient groups included pregnancy, lactation, presence of autoimmune or systemic inflammatory disorders, and history of recent infection. The healthy control group consisted of women with no personal history of breast disease, systemic inflammatory disorders, or malignancy.

Participants were enrolled consecutively from a single tertiary referral center over the study period. Although no formal matching procedure was implemented, group sizes were kept comparable. The relatively younger age profile of the BC cohort and the higher frequency of HER2-positive cases likely reflect center-specific referral patterns and the limited sample size, rather than the epidemiological distribution of the general BC population.

### 2.3. Exclusion Criteria

Exclusion criteria for both patient groups included pregnancy, lactation, autoimmune or systemic inflammatory diseases, and recent infection. Patients were further excluded if histopathological evaluation revealed alternative causes of granulomatous inflammation. These included infectious etiologies (e.g., tuberculosis, fungal or parasitic infections), systemic granulomatous diseases (e.g., sarcoidosis, granulomatosis with polyangiitis), and non-infectious causes such as foreign body reactions.

The healthy control group consisted of women with no history of breast disease, malignancy, or systemic inflammatory conditions.

### 2.4. Clinical and Laboratory Data Collection

Demographic data, body mass index (BMI), and clinicopathological characteristics [tumor localization, histological grade, estrogen receptor (ER), progesterone receptor (PR), HER2/neu status, and Ki-67 proliferation index for BC patients] were recorded. Blood samples were collected from all participants after overnight fasting. Serum was separated and stored at −80 °C until analysis.

Serum concentrations of α-defensin 1, β-defensin 1, and β-defensin 2 were quantified using commercially available enzyme-linked immunosorbent assay (ELISA) kits (Cloud-Clone Corp., Wuhan, China). All assays were performed in duplicate according to the manufacturer’s protocols, and both intra-assay and inter-assay coefficients of variation were <10%.

Serum tumor markers, including CEA, CA15-3, CA19-9, and CA125, were quantified using an electrochemiluminescence immunoassay (ECLIA). CRP was determined by an immunoturbidimetric method, and white blood cell (WBC) counts were obtained from complete blood count analyses using an automated hematology analyzer.

### 2.5. Statistical Analysis

Statistical analyses were performed using SPSS v21.0 (IBM Corp., Armonk, NY, USA). Continuous variables were expressed as mean ± SD and categorical variables as counts and percentages. Normality was assessed with the Shapiro–Wilk test. Group comparisons (BC, IGM, and controls) were conducted using one-way ANOVA or Kruskal–Wallis tests with post hoc analyses. Categorical associations were examined by chi-square or Fisher’s exact tests. ROC curve analysis evaluated the diagnostic performance of serum α-defensin 1, β-defensin 1, and β-defensin 2, with sensitivity, specificity, and AUC (95% CI) reported. Logistic regression models assessed independent predictive value: Model 1 included defensins only, while Model 2 added tumor markers and clinical covariates. Results were expressed as ORs with 95% CI. Correlations between defensins and inflammatory markers were analyzed using Pearson coefficients (weak: r < 0.3; moderate: 0.3–0.6; strong: ≥0.6) and visualized with scatter plots. A *p*-value < 0.05 was considered statistically significant.

## 3. Results

A total of 150 participants were included: 50 with IGM, 50 with BC, and 50 healthy controls. The mean age was similar across groups (*p* = 0.773). Body mass index (BMI) was significantly higher in the BC group compared to IGM and controls (*p* < 0.001). Among tumor markers, CA19-9, CA15-3, and CA125 levels were significantly elevated in the IGM and BC groups compared to healthy controls (all *p* < 0.001), whereas CEA showed no significant differences (*p* = 0.093). Inflammatory markers, including CRP and leukocyte counts, were also significantly higher in the patient groups compared to healthy individuals (both *p* < 0.001) (Table 1).

**Figure 1:**
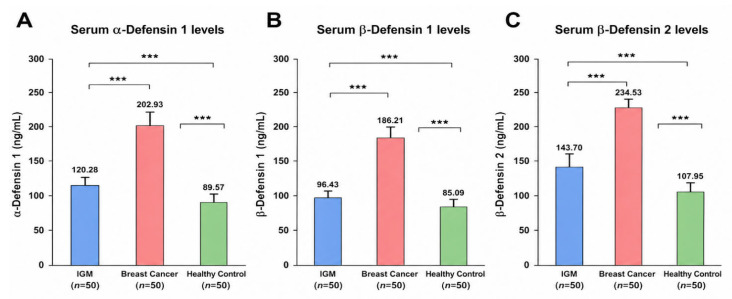
Serum defensin levels in the study groups: (**A**) α-defensin 1, (**B**) β-defensin 1, and (**C**) β-defensin 2 in idiopathic granulomatous mastitis (IGM), breast cancer, and healthy controls. Data are presented as mean ± SD. *** *p* < 0.001 (one-way ANOVA with Tukey’s post hoc test).

Boxplot analysis revealed that serum α-Defensin 1, β-Defensin 1, and β-Defensin 2 levels were significantly elevated in the BC group compared with both IGM patients and healthy controls (all *p* < 0.001) (Figure 1). While α-defensin 1 and β-Defensin 1 showed partial overlaps between IGM and control groups, their median levels remained consistently higher in BC. In contrast, β-Defensin 2 demonstrated the most pronounced discriminatory capacity, with BC patients exhibiting markedly elevated concentrations and minimal distributional overlap with either comparator group. These findings underscore β-Defensin 2 as the most robust defensin biomarker in differentiating BC from benign inflammatory conditions and healthy individuals.

The clinicopathological features of the BC group are shown in Table 2. In the BC cohort, invasive ductal carcinoma represented the predominant histological type, accounting for 72% of cases, while 28% had mixed histology. Tumor localization was nearly balanced, with 48% occurring in the right breast and 52% in the left. Most patients were classified as histologic grade II (68%), with the remaining 32% as grade III. Molecular analysis revealed that 70% and 68% of patients were positive for estrogen and progesterone receptors, respectively, while HER2/neu was overexpressed in 44%. The mean Ki67 proliferation index was 22.9 ± 17.9%, indicating considerable heterogeneity in tumor proliferation rates within the cohort.

### 3.1. ROC Analysis

**Figure 2:**
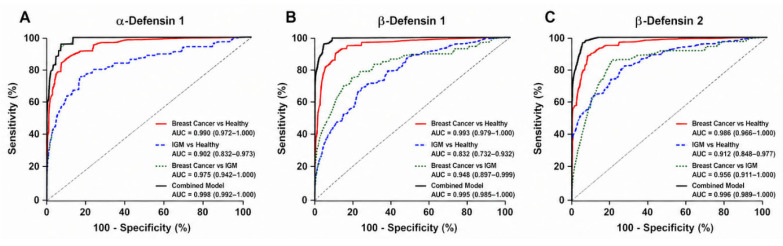
ROC curves of defensins and the combined model for differentiating breast cancer, idiopathic granulomatous mastitis, and healthy controls. (**A**) α-Defensin 1; (**B**) β-Defensin 1; (**C**) β-Defensin 2.

ROC analysis demonstrated that defensins exhibited high diagnostic accuracy, with AUC values ranging from 0.95 to 0.99 (Table 3). Sensitivity and specificity values were consistently above 89%, indicating strong and reliable diagnostic performance. Logistic regression analysis (Table 4) revealed balanced odds ratios (approximately 1.08–1.15) with corresponding coefficients, showing that defensins were significantly associated with disease status. Among the markers, β-defensin 2 showed the strongest association, while the combined defensin model achieved the highest AUC (approximately 0.99). Figure 2 illustrates the ROC curves of individual defensins and the combined model, demonstrating that the integration of markers improved diagnostic classification compared to single analytes.

**Figure 3:**
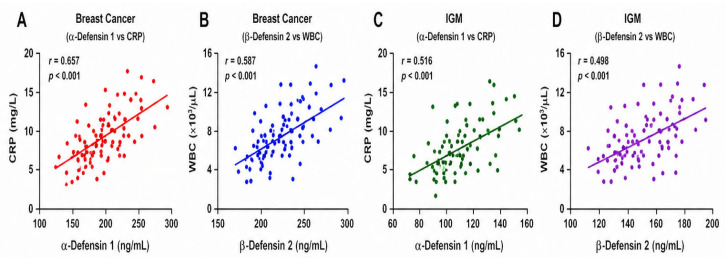
Correlation plots of defensins with inflammatory markers. (**A**) Breast cancer (α-Defensin 1 vs. CRP). (**B**) Breast cancer (β-Defensin 2 vs. WBC). (**C**) IGM (α-Defensin 1 vs. CRP). (**D**) IGM (β-Defensin 2 vs. WBC).

Correlation analyses demonstrated significant and moderate positive associations between defensins and inflammatory markers (Figure 3). In the BC group, α-Defensin 1 correlated moderately with CRP (r ≈ 0.65, *p* < 0.001), while β-Defensin 2 showed a moderate-to-strong correlation with WBC counts (r ≈ 0.59, *p* < 0.001). In the IGM group, α-Defensin 1 levels were positively associated with CRP (r ≈ 0.51, *p* < 0.001), and β-Defensin 2 correlated with WBC counts (r ≈ 0.50, *p* < 0.001).

### 3.2. Stepwise Logistic Regression Analysis

Stepwise forward logistic regression initially considered defensins and subsequently tumor markers. Interestingly, α- and β-defensins were individually strong predictors in univariate models but, due to multicollinearity, were not retained together in the final multivariable model. Instead, the algorithm selected CA15-3 and CA125 as the most parsimonious independent predictors of BC versus idiopathic granulomatous mastitis (IGM).

**Figure 4:**
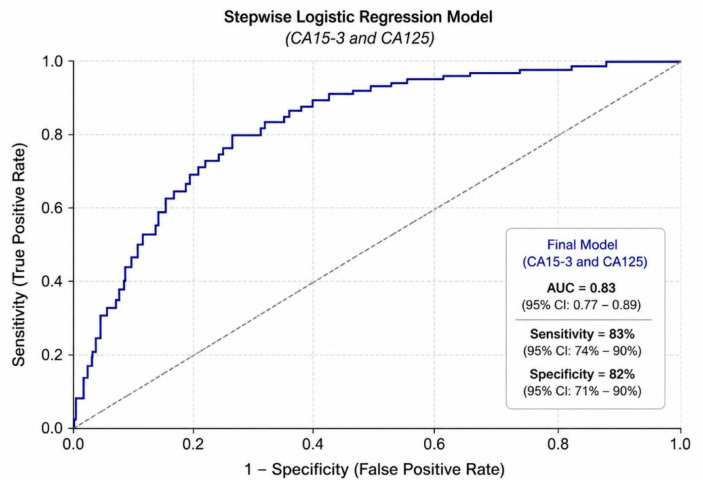
Receiver operating characteristic (ROC) curve of the final logistic regression model including CA15-3 and CA125 for distinguishing breast cancer from idiopathic granulomatous mastitis, showing good diagnostic performance (AUC = 0.83).

The ROC curve of the final logistic regression model is presented in Figure 4 (AUC = 0.83).

### 3.3. Logistic Regression Models

In the defensin-only model, all defensins were independently associated with disease status. Specifically, each unit increase in α-defensin 1, β-defensin 1, and β-defensin 2 was associated with increased odds of BC, with odds ratios (ORs) of 1.08, 1.12, and 1.15, respectively (Table 4).

In the tumor marker model, both CA15-3 and CA125 were significantly associated with BC. Each unit increase in CA15-3 and CA125 increased the odds of BC relative to IGM (OR = 1.08, 95% CI: 1.05–1.11, *p* < 0.001; and OR = 1.07, 95% CI: 1.04–1.10, *p* < 0.001, respectively) (Table 5).

### 3.4. Model Interpretation

Overall, while defensins demonstrated strong predictive value in univariate and single-marker models, tumor markers—particularly CA15-3 and CA125—provided a more stable and parsimonious multivariable model. These findings suggest that tumor markers outperform defensins in distinguishing BC from IGM when considered jointly, while defensins may still have complementary diagnostic value.

## 4. Discussion

This study demonstrates that serum defensins, particularly β-defensin 2, are significantly elevated in patients with BC compared with IGM and healthy controls. Among the evaluated biomarkers, β-defensin 2 exhibited the strongest discriminatory capacity, and the combined biomarker model further improved diagnostic performance. However, although defensins showed significant associations in univariate analyses, they were not retained as independent predictors in multivariable models. In contrast, conventional tumor markers such as CA15-3 and CA125 remained independent predictors, indicating that defensins may serve as adjunctive rather than standalone diagnostic biomarkers.

Defensins are multifunctional peptides with established roles in innate immunity as well as emerging functions in tumor biology [6,7,8,10]. Experimental studies have demonstrated that defensins can exert both tumor-promoting and tumor-suppressive effects depending on the biological context. Β-defensin 2 has been shown to modulate immune responses through activation of macrophages, enhancement of cytokine signaling, and stimulation of both innate and adaptive immune pathways [11,12,13,14,15]. These immunomodulatory properties are consistent with our findings of elevated β-defensin 2 levels in BC, suggesting a link between defensin expression and tumor-associated immune activation.

Importantly, the significantly higher defensin levels observed in BC compared with IGM indicate that their elevation cannot be explained solely by non-specific inflammation. While IGM represents a predominantly inflammatory condition, the more pronounced increase in defensins in BC suggests the contribution of additional tumor-related mechanisms. Circulating defensin levels may therefore reflect a composite signal integrating systemic inflammation and tumor–immune interactions rather than purely local inflammatory processes.

The role of α-defensin 1 as a marker of inflammatory activity has been well documented. Sobti et al. [16] demonstrated a correlation between α-defensin 1 levels and infection severity in implant-based breast reconstruction, while multicenter studies by Basta et al. [17,18] showed that α-defensin 1 outperformed conventional culture methods in diagnosing periprosthetic breast infections. Our findings extend these observations by demonstrating that α-defensin 1 is elevated in both malignant and inflammatory breast conditions, supporting its role as a general marker of immune activation across diverse breast pathologies.

Defensins also possess structural and functional properties relevant to cancer biology. Human β-defensin 1 is characterized by a disulfide-stabilized structure and exhibits antimicrobial, antifungal, and antitumor activity [19]. Reduced expression of β-defensin 1 has been reported in malignant tissues, and restoration of its expression has been associated with tumor suppression in experimental models [20,21,22]. In addition, recent studies have suggested that restoration of β-defensin 1 may enhance the efficacy of chemotherapy, highlighting its potential as a therapeutic target [23]. These findings support the concept that defensins may play dual roles as both biomarkers and modulators of tumor behavior.

Similarly, β-defensin 2 has been shown to exert broad immunomodulatory effects, including regulation of cytokine production, immune cell proliferation, and angiogenesis [13,24]. In addition, recent studies have suggested that restoration of β-defensin 1 may enhance the efficacy of chemotherapy, highlighting its potential as a therapeutic target [23,24,25]. Experimental studies have demonstrated that β-defensin 2 can enhance antitumor immunity through activation of T cells and natural killer cells [15,26]. In line with these findings, the elevated β-defensin 2 levels observed in our cohort may reflect not only systemic inflammation but also active involvement in tumor–immune interactions.

BC is a biologically heterogeneous disease with distinct molecular subtypes characterized by different immune profiles. Estrogen receptor-positive tumors are generally considered less inflamed compared with HER2-positive or triple-negative subtypes. However, the consistently elevated defensin levels observed across the BC cohort suggest that circulating defensins may reflect systemic immune activation rather than local tumor microenvironment characteristics alone. This may explain their elevation even in subtypes typically associated with lower inflammatory activity. Additionally, potential enrichment of specific BC subtypes, such as HER2-positive tumors, may influence defensin expression due to differences in tumor biology and immune activation, which should be considered when interpreting the generalizability of these results.

The correlation analyses in this study were limited to CRP and total leukocyte counts, which represent general markers of systemic inflammation but do not fully capture the complexity of immune responses. The absence of leukocyte subtype analysis and tumor microenvironment evaluation limits mechanistic interpretation. Therefore, the observed correlations should be interpreted cautiously as indicators of systemic immune activation rather than specific immunological pathways.

From a clinical perspective, defensins are not intended to replace histopathological evaluation, which remains the gold standard for diagnosis. Instead, they may serve as adjunctive biomarkers in selected clinical scenarios, particularly in patients with overlapping clinical and radiological findings where differentiation between IGM and BC is challenging. In this context, defensins should be considered complementary tools that may enhance, but not replace, established diagnostic approaches. In such cases, a non-invasive biomarker panel may support pre-diagnostic risk stratification and reduce diagnostic uncertainty. The improved performance observed with combined biomarker models further supports a multiplex diagnostic approach.

This study has several strengths, including its comparative design incorporating malignant, inflammatory, and healthy groups, and the integrated evaluation of defensins alongside conventional biomarkers using standardized methods and advanced statistical analyses.

However, several limitations should be acknowledged. This study’s single-center, cross-sectional design and relatively small sample size may limit generalizability. The absence of subtype-specific analyses and detailed immune profiling restricts biological interpretation. Importantly, defensins did not remain independent predictors in multivariable models, reinforcing that their clinical utility may be optimized when used in combination with established markers rather than as standalone diagnostic tools.

## 5. Conclusions

In conclusion, this study demonstrates that both defensins and tumor markers have diagnostic value in distinguishing breast cancer (BC) from idiopathic granulomatous mastitis (IGM). While defensins showed strong discriminatory ability in univariate analyses, their role in multivariable models was limited due to multicollinearity. In contrast, tumor markers—particularly CA15-3 and CA125—emerged as robust and independent predictors, providing a more stable and parsimonious model with good diagnostic performance. The combined logistic regression model based on CA15-3 and CA125 achieved satisfactory discriminative accuracy, highlighting their clinical relevance in differentiating BC from IGM. These findings indicate that defensins should be considered adjunctive biomarkers that may complement, but not replace, established tumor markers and histopathological evaluation. Overall, these findings should be considered exploratory and hypothesis-generating. Further validation in larger, multicenter, and prospective studies is required before clinical implementation.

## Figures and Tables

**Table 1:** Demographic characteristics and tumor markers of breast cancer (BC), idiopathic granulomatous mastitis (IGM), and healthy controls.

	BC (*n* = 50)	IGM (*n* = 50)	Healthy Controls (*n* = 50)	*p*-Value
Age (years)	44.30 ± 7.55	43.89 ± 6.53	44.92 ± 8.02	0.773
BMI (kg/m^2^)	28.19 ± 5.66	26.39 ± 4.43	27.08 ± 5.30	<0.001
CEA (ng/mL)	2.05 ± 0.83	1.73 ± 0.81	1.77 ± 0.74	0.093
CA19-9 (U/mL)	17.00 ± 11.81	23.29 ± 19.79	8.19 ± 3.43	<0.001
CA15-3 (U/mL)	22.45 ± 13.30	19.01 ± 6.81	10.93 ± 5.23	<0.001
CA125 (U/mL)	15.38 ± 8.09	27.73 ± 15.34	12.88 ± 7.43	<0.001
CRP (mg/L)	6.28 ± 2.17	8.2 ± 2.10	3.22 ± 0.83	<0.001
Leukocyte (×10⁹/L)	7.70 ± 2.10	8.7 ± 2.30	5.94 ± 1.78	<0.001
α-Defensin 1 (ng/mL)	202.93 ± 22.22	120.28 ± 13.84	89.57 ± 8.73	<0.001
β-Defensin 1 (ng/mL)	186.21 ± 15.29	96.43 ± 10.72	85.09 ± 9.32	<0.001
β-Defensin 2 (ng/mL)	234.53 ± 21.20	143.70 ± 20.80	107.95 ± 9.57	<0.001

Values are presented as mean ± standard deviation. *p*-values were calculated using one-way ANOVA for continuous variables across the three groups. Post hoc analyses were performed using Tukey’s test. A *p*-value < 0.05 was considered statistically significant. BMI—Body Mass Index; CEA—Carcinoembryonic Antigen; CA19-9—Carbohydrate Antigen 19-9; CA15-3—Cancer Antigen 15-3; CA125—Cancer Antigen 125; CRP—C-Reactive Protein.

**Table 2:** Clinicopathological characteristics of the breast cancer cohort (*n* = 50).

Variable	Value
Ki67 (%)	22.9 ± 17.9
Cancer type	IDC: 36 (72%); Mixed: 14 (28%)
Localization	Right: 24 (48%); Left: 26 (52%)
Histological grade	Grade II: 34 (68%); Grade III: 16 (32%)
Estrogen receptor (ER)	Positive: 35 (70%); Negative: 15 (30%)
Progesterone receptor (PR)	Positive: 32 (64%); Negative: 18 (36%)
HER2/neu	Positive: 22 (44%); Negative: 28 (56%)

IDC—invasive ductal carcinoma.

**Table 3:** ROC Analysis of Defensins and the Combined Ridge Model.

Variable	Cutoff	Sensitivity (%)	Specificity (%)	AUC (95% CI)
α-Defensin 1	148.5	0.92	0.90	0.97 (0.94–0.99)
β-Defensin 1	128.0	0.90	0.89	0.95 (0.91–0.98)
β-Defensin 2	178.0	0.91	0.90	0.96 (0.93–0.99)
Combined Ridge Model	–	0.95	0.94	0.99 (0.97–1.00)

**Table 4:** Logistic Regression Analysis of Defensins.

Variable	Coefficient (β)	Odds Ratio (OR)	95% CI	*p*-Value
α-Defensin 1	0.077	1.08	1.04–1.12	<0.001
β-Defensin 1	0.113	1.12	1.07–1.17	<0.001
β-Defensin 2	0.140	1.15	1.09–1.21	<0.001

**Table 5:** Logistic regression analysis demonstrates the association between tumor markers and breast cancer. A per-unit increase in CA15-3 and CA125 significantly increased the odds of breast cancer compared to IGM.

Variable	β (Coefficient)	Odds Ratio (OR)	95% CI	*p*-Value
CA15-3	0.081	1.08	1.05–1.11	<0.001
CA125	0.063	1.07	1.04–1.10	<0.001
Constant	−10.90	–	–	<0.001

## Data Availability

The datasets used and/or analyzed during the current study are available from the corresponding author on reasonable request.
